# Cerebroventricular Microinjection (CVMI) into Adult Zebrafish Brain Is an Efficient Misexpression Method for Forebrain Ventricular Cells

**DOI:** 10.1371/journal.pone.0027395

**Published:** 2011-11-04

**Authors:** Caghan Kizil, Michael Brand

**Affiliations:** DFG-Center for Regenerative Therapies Dresden, Cluster of Excellence (CRTD), and Biotechnology Center, Technische Universität Dresden, Dresden, Germany; Center for Regenerative Therapies Dresden, Germany

## Abstract

The teleost fish *Danio rerio* (zebrafish) has a remarkable ability to generate newborn neurons in its brain at adult stages of its lifespan-a process called adult neurogenesis. This ability relies on proliferating ventricular progenitors and is in striking contrast to mammalian brains that have rather restricted capacity for adult neurogenesis. Therefore, investigating the zebrafish brain can help not only to elucidate the molecular mechanisms of widespread adult neurogenesis in a vertebrate species, but also to design therapies in humans with what we learn from this teleost. Yet, understanding the cellular behavior and molecular programs underlying different biological processes in the adult zebrafish brain requires techniques that allow manipulation of gene function. As a complementary method to the currently used misexpression techniques in zebrafish, such as transgenic approaches or electroporation-based delivery of DNA, we devised a cerebroventricular microinjection (CVMI)-assisted knockdown protocol that relies on vivo morpholino oligonucleotides, which do not require electroporation for cellular uptake. This rapid method allows uniform and efficient knockdown of genes in the ventricular cells of the zebrafish brain, which contain the neurogenic progenitors. We also provide data on the use of CVMI for growth factor administration to the brain – in our case FGF8, which modulates the proliferation rate of the ventricular cells. In this paper, we describe the CVMI method and discuss its potential uses in zebrafish.

## Introduction

The adult zebrafish brain contains ventricular progenitor cells that sustain adult neurogenesis [1]–[6]. Understanding the genes and molecular programs involved in these progenitors require analyses of gene function. Different transgenic approaches using simple promoter fusions to reporter genes, Cre/lox-based dual transgenic systems [7]–[11], Tet on-off strategy [12]–[14] and Gal4-UAS-based conditional expression techniques [15]–[18] or focal injection of DNA followed by electroporation [19] serve as valuable tools for misexpression purposes. However, a uniform and efficient targeting is not easy to achieve due to several reasons such as transgenes can be prone to silencing in adult zebrafish [20], [21] or the entire set of enhancer elements for regionally restricted expression may not be available. Similarly, focal injections and electroporation cause mosaic targeting, which may complicate the functional interpretations of experiments in which a broader misexpression is needed. In zebrafish and several other vertebrate model organisms, morpholino-based inactivation has proven to be a rapid and often reliable way to study gene function [22]–[28]. Several studies have also successfully used morpholinos and subsequent electroporation in adult fish tissues such as the caudal fin or retina [29]–[31]. However, electroporation may have an adverse effect on the viability of cells [32], [33]. Whereas, vivo morpholinos are new tools for knockdowns, they do not require electroporation for delivery into tissues [26], [34], [35] and were recently used successfully in the adult zebrafish [36], [37]. Therefore, we established for the first time in the adult zebrafish brain a vivo morpholino oligonucleotide-based cerebroventricular microinjection (CVMI) method for gene knockdowns. After the injection through a skull incision in one place-in this paper above the optic tectum-CVMI can be used to target cells uniformly at or near the site of injection, in our case in the ventricular zone of the forebrain, which contains neurogenic progenitor cells [6]. CVMI of vivo morpholinos blocks protein production efficiently in a dose-dependent manner and within few cell diameters along the ventricular surface for approximately up to a week without overt toxicity. We also showed that knockdowns using CVMI is sufficient to cause functional consequences; for instance, by knocking down endogenous proliferating cell nuclear antigen (PCNA) and BrdU pulse-chase experiments, we show that production of newborn neurons reduces significantly in adult zebrafish brains. We found that the immediacy of maximum knockdown (70–90%) varies for different genes possibly due to the stability and the levels of the proteins in the cell, while we achieved more than 50% knockdown for PCNA within 12 hours of injection. We attained the maximum efficiency of knockdown at 3 days post injection (dpi) for mCherry fluorescent reporter, whereas 1 day was enough for knocking down PCNA by 80–90%.

In overall, CVMI method (1) allows analyzing endogenous genes in ventricular cells that contain the progenitors for adult neurogenesis, (2) provides an easy way of delivery since it eliminates the electroporation step, (3) results in a uniform and rapid knockdown, and (4) has the potential to serve as a complementary approach to the existing tools of misexpression in the adult zebrafish brain. We also report that CVMI is useful for injecting proteins as well, such as growth factors to modulate the cellular behavior of the ventricular cells. We showed that ventricular cell proliferation increases after CVMI of FGF8, which was previously shown by transgenic overexpression to be a mitogenic signal [5]. Based on our results, we argue that with these features, CVMI serves as a reliable and rapid way of uniform misexpression using morpholinos and proteins and will help conducting functional studies in the adult zebrafish brain.

## Results and Discussion

### CMTPX-Red injection labels ventricular and periventricular cells

**Figure 1:**
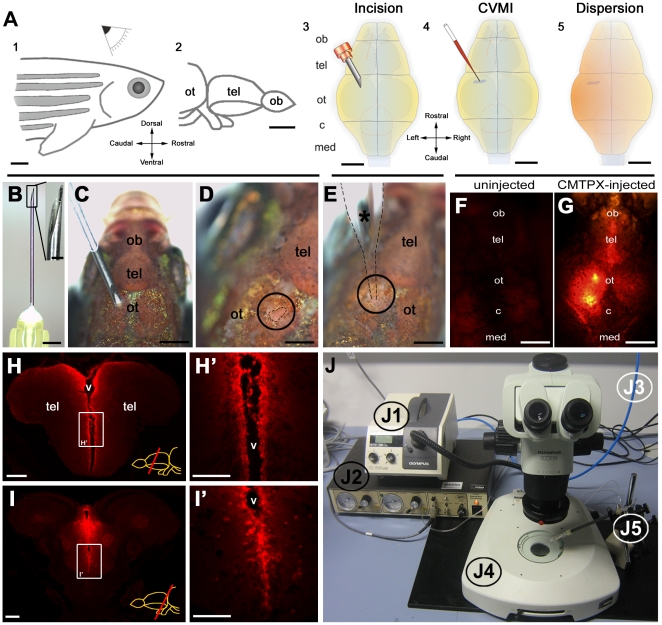
Overview of cerebroventricular microinjection (CVMI) paradigm and its target regions. (A) CVMI is performed at the dorsal surface of the head (1) and it targets, in this example, the forebrain that is rostral to the optic tectum (2). For injection, an incision is made into the skull over the optic tectum using a barbed-end canula (3). Through this slit, liquid is injected using a glass capillary (4). Injected liquid disperses rostrally (5). (B) The canula used for incision. (C) The incision on an adult fish. Dorsal view. (D) The incision site marked by dashed lines. (E) Injection with the glass capillary (*) (dotted lines mark the outline). (F) Dorsal view of an uninjected adult zebrafish head in red fluorescence channel. (G) Dorsal view of a CMTPX-injected adult zebrafish head in red fluorescence channel. (H) Cross section through the telencephalon. Ventricular cells are labelled with CMTPX. (H') Higher magnification of the box in H. (I) Cross section through the midbrain. Ventricular cells are labelled with CMTPX. (I') Higher magnification of the box in I. (J) Injection apparatus. J1: halogen light source with ring illuminator. J2: vacuum pump for microinjection. J3: pressurized air source. J4: dissecting microscope. J5: injection holder, needle and tubing. Scale bars: 500 µm A–G, 100 µm H-I'. ot: optic tectum, tel: telencephalon, ob: olfactory bulb, c: cerebellum, med: medulla, v: ventricle.

We developed a cerebroventricular microinjection (CVMI) method for adult zebrafish brain, which is suitable for functional studies such as gene knock-down and agonist/antagonist-based manipulation of signal transduction (Fig. 1). This method relies on dispersion of the injected liquid throughout the cerebroventricular fluid and uptake by cells close to the ventricle. In order to estimate the extent of dispersion and the uptake of the injected liquid, we used a cell tracker dye (CMTPX-Red, Molecular Probes) (Fig. 1). CMTPX itself is a non-fluorescent cell-permeant molecule that, upon internalization by the cells, is converted by Gluthatione-S-transferase to a red fluorescent compound [38]. Therefore, the fluorescence is an indication of the cells that take up the injected liquid and not of the residual dye that is in the cerebroventricular fluid.

**Figure 2:**
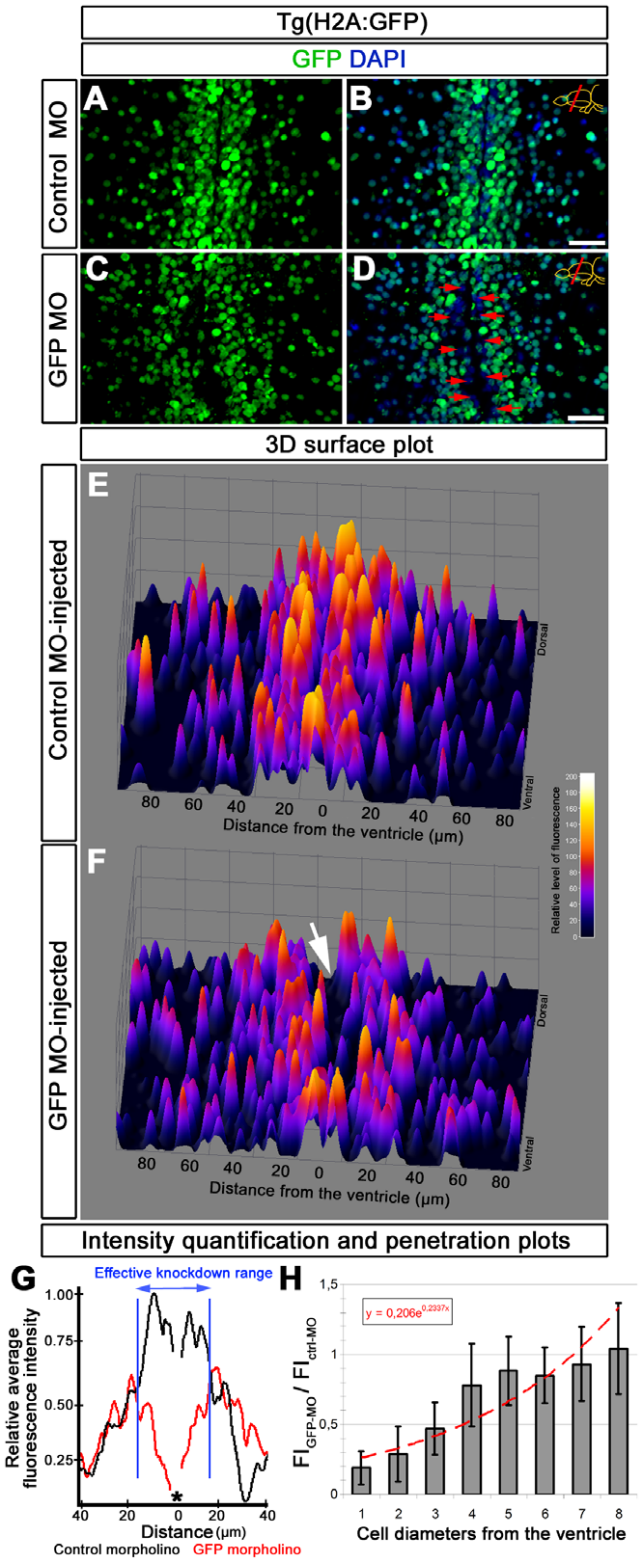
Analysis of morpholino penetration and efficient knockdown range. (A) GFP immunohistochemistry (IHC) on control morpholino-injected (Ctrl-MO) brain sections. (B) DAPI colocalization of A. (C) GFP immunohistochemistry (IHC) on GFP antisense morpholino-injected (GFP-MO) brain sections. (D) DAPI colocalization of C. Red arrows exemplify fully knocked-down cells. (E) 3D surface plot of A. x-axis: distance from the ventricle; y-axis: ventral to dorsal orientation; z-axis: relative level of fluorescence intensity. (F) 3D surface plot of C. x-axis: distance from the ventricle; y-axis: ventral to dorsal orientation; z-axis: relative level of fluorescence intensity. White arrow shows the gap, which indicates the knocked down cells. (G) Intensity quantification plot depicting relative fluorescence intensity versus distance from the ventricle in Ctrl-MO (black lines) and GFP-MO (red lines) injections. Black asterisk indicates the ventricle. Blue bars delineate the borders where GFP-MO injection leads to overt reduction in GFP fluorescence intensity. (H) Penetration prediction graph. The ratio of fluorescence intensity values between Ctrl-MO (FI_Ctrl-MO_) and GFP-MO (FI_GFP-MO_) versus cell diameters away from the ventricle is sketched. The trendline indicates the extent of knockdown in relation to cell diameters. Scale bars 20 µm.

When we sacrificed the fish 10 minutes after the injection and sectioned the brains, we observed CMTPX-Red-positive cells along the ventricular regions of the brain (Fig. 1G-1I'), indicating that the cells lining the ventricle are targeted effectively (Fig. 1H' and 1I'). The fluorescent cells are on average within few cell diameters of the ventricular surface (Fig. 2H' and 2I'). These results show that CVMI can effectively target cells in close proximity to the ventricle, while the deep parenchymal cells are not accessible with this technique.

### Vivo morpholinos exert efficient knockdown within few cells diameters from the ventricle after CVMI

In order to determine how far vivo morpholinos can penetrate into the tissue after CVMI, we injected GFP antisense morpholinos to *Tg(H2A:GFP)* transgenic line that ubiquitously labels the cells with GFP fluorescence reporter. At 2 days after injection, compared to the control morpholino-injected brains, (Fig. 2A, 2B, 2E), GFP antisense morpholino injection knocks down fluorescence reporter in the cells close to the ventricle (Fig. 2C, 2D, 2F). In order to determine the efficient knockdown range, we plotted the histogram of fluorescence intensity relative to ventricular location (Fig. 2G). We observed that on average within 17.4 µm away from the ventricle, GFP morpholino injection leads to significant reduction in average fluorescence intensity (Fig. 2G). To analyze the extent of knockdown with CVMI, we determined the average fluorescence intensity of GFP morpholino-injected (FI_GFP-MO_) and control morpholino-injected (FI_Ctrl-MO_) brains at every cell diameter away from the ventricle (Fig. 2G). Based on the fitting curve, we found that 50% reduction in fluorescence intensity after CVMI of vivo morpholinos could be achieved within 3.79 cell diameters (Fig. 2G). Our results also indicate that the CVMI of vivo morpholinos is not toxic to cells of the brain and does not lead to cell death determined by TUNEL staining (data not shown).

### Knockdown efficiency and immediacy with vivo morpholinos is peculiar to the protein analyzed

**Figure 3:**
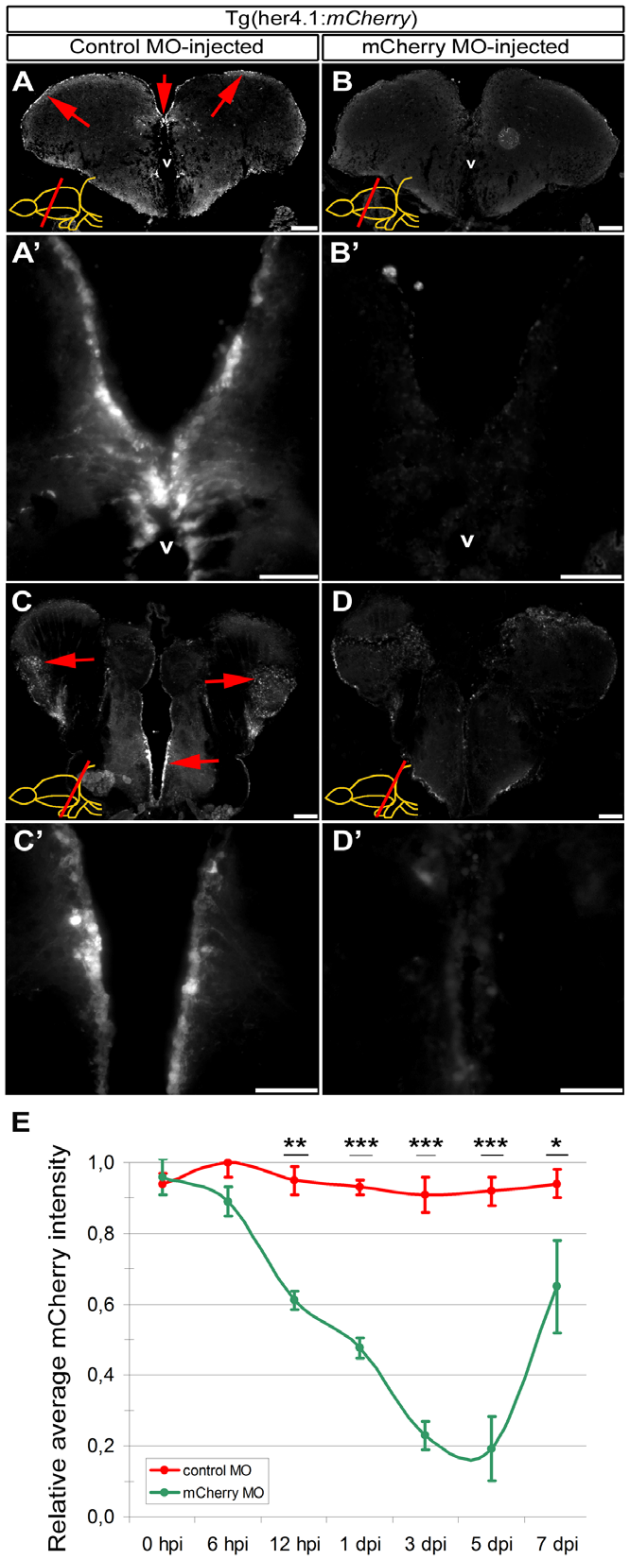
Morpholino-mediated gene knockdown using CVMI in *Tg(her4.1:mCherry)* reporter line. (A) Fluorescence reporter activity of *Tg(her4.1:mCherry)* transgenic line in the radial glial cells (red arrows along the ventricular surface) of the rostral telencephalon. (A') Higher magnification of the dorsomedial region of A. (B) Fluorescence reporter activity in rostral telencephalon injected with translation-blocking vivo morpholinos for mCherry transgene. (B') Higher magnification of the dorsomedial region of B, indicating the significant reduction of reporter activity. (C) Fluorescence reporter activity of *Tg(her4.1:mCherry)* transgenic line in the radial glial cells (red arrows along the ventricular surface) of the caudal telencephalon. (C') Higher magnification of the medial region of C. (D) Fluorescence reporter activity in caudal telencephalon injected with translation-blocking vivo morpholinos for mCherry transgene. (D') Higher magnification of the medial region of D, indicating the significant reduction of reporter activity. (E) Graph depicts the average mCherry fluorescence intensity in mCherry antisense morpholino-injected brains (green line) over a time course relative to control morpholino-injected brains (red line). Scale bars 50 µm. v: ventricle, tel: telencephalon, hpi: hours post injection, dpi: days post injection. N = 6 adult fish for each time point.

Since CVMI can target ventricular cells that contain radial glial (RG) progenitor cells [2], [3], [5], [6], [39], [40], we examined whether gene expression in the RG can be knocked-down using morpholino oligonucleotides. For this purpose, we used a transgenic line that drives the expression of fluorescent mCherry protein in RG cells (*Tg(her4.1:mCherry*)) (Fig. 3A, 3A', 3C, 3C') and injected 500 picomoles of mCherry translation-blocking vivo morpholinos (500 µM, 1 µl). This injection leads to significant reduction of fluorescence reporter activity at the ventricular regions (Fig. 3B, 3B', 3D, 3D'; 3 day-post-injection (dpi) is shown).

To determine the efficiency and temporal dynamics of knockdowns, we dissected the brains, cryosectioned and measured the mCherry fluorescence intensity on every section at different time points (Fig. 3E). We observed the first statistically significant reduction of fluorescence intensity at 12 hours post injection (35.6±3.2%). Knockdown is more pronounced at later stages (48.7±4.1% at 1 dpi, 74.7±3.9% at 3 dpi and 79.1±5.2% at 5 dpi). The effect of morpholino knock-down reduces later than 5 dpi (30.8±9.1% at 7 dpi) and fluorescence levels return back to original levels at around 10 dpi (data not shown). These results indicate that morpholino injection using CVMI can knockdown the gene expression in the RG cells of the forebrain and that the statistically significant knockdown period with mCherry antisense vivo morpholinos for mCherry reporter line is between 12 hpi and 7 dpi; more than 50% knockdown is between 1 and 5 dpi; and the maximum efficiency is between 3 and 5 dpi.

**Figure 4:**
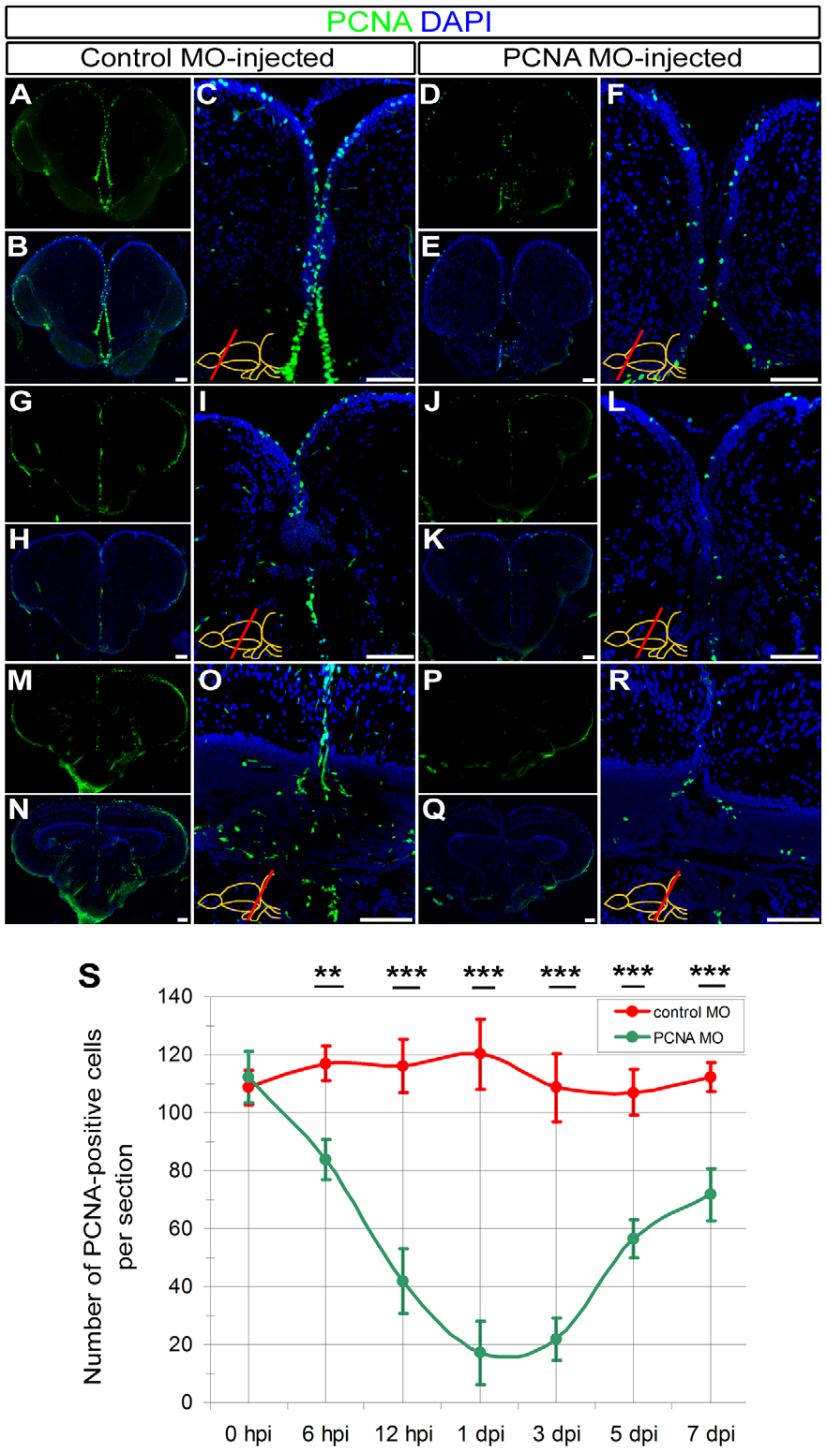
Knocking-down PCNA as an endogenous gene using CVMI. (A) PCNA immunohistochemistry (IHC) on rostral telencephalon of control morpholino-injected (ctrl-MO) brains. (B) DAPI counterstaining on A. (C) High magnification image of medial ventricular region of B. (D) PCNA IHC on rostral telencephalon of PCNA morpholino-injected (PCNA-MO) brains. (E) DAPI counterstaining on D. (F) High magnification image of medial ventricular region of E. (G) PCNA IHC on rostral telencephalon of ctrl-MO brains. (H) DAPI counterstaining on G. (I) High magnification image of medial ventricular region of H. (J) PCNA IHC on telencephalon of PCNA-MO brains on a more caudal level. (K) DAPI counterstaining on J. (L) High magnification image of medial ventricular region of K. (M) PCNA IHC on rostral optic tectum of ctrl-MO brains. (N) DAPI counterstaining on M. (O) High magnification image of dorsal region of N. (P) PCNA IHC on rostral optic tectum of PCNA-MO brains. (Q) DAPI counterstaining on P. (R) High magnification image of dorsal region of Q. (S) Graph depicts the average number of PCNA-positive cells in PCNA antisense morpholino-injected brains (green line) over a time course relative to control morpholino-injected brains (red line). Scale bars 50 µm. hpi: hours post injection, dpi: days post injection. N = 3 adult fish for each time point.

Since the expression level of a gene in cells is an important determinant of how well the knockdown can be achieved with a given concentration, we also knocked down PCNA as an endogenous gene in ventricular cells (Fig. 4). We observed that PCNA antisense morpholino reduces the number of PCNA-positive cells significantly (Fig. 4A–4R). We observed the first significant reduction of PCNA-positive cells at 6 hours post injection (30.5±5.6%). Knockdown efficiency increases at later stages (55.3±8.6% at 12 hpi, 84.2±9.7% at 1 dpi, 80.1±5.0% at 3 dpi) (Fig. 4S). After 3 dpi, the effect reduces; for instance at 5 dpi, the efficiency is 47.3±10.3%, and there is still 37.5±8.8% reduction in PCNA-positive cells at 7 dpi. These results indicate that the statistically significant knockdown period for PCNA antisense morpholinos after CVMI is between 6 hpi and 7 dpi; more than 50% knockdown is between 12 hpi and 5 dpi; and the maximum efficiency is between 1 and 3 dpi. These results indicate that (1) endogenous genes can be knocked down with CVMI, (2) the immediacy, efficient knockdown period and maximum efficiency of knockdowns varies with the protein of interest, and (3) CVMI can knockdown genes in a statistically significant manner until 7 dpi compared to control morpholino injections. Since the effects of knockdown are different at every time point after injection, the right time point that provides enough blockage for any functional consequence of interest has to be determined by the experimenter.

### Knocking down PCNA results in reduced neurogenesis response

**Figure 5:**
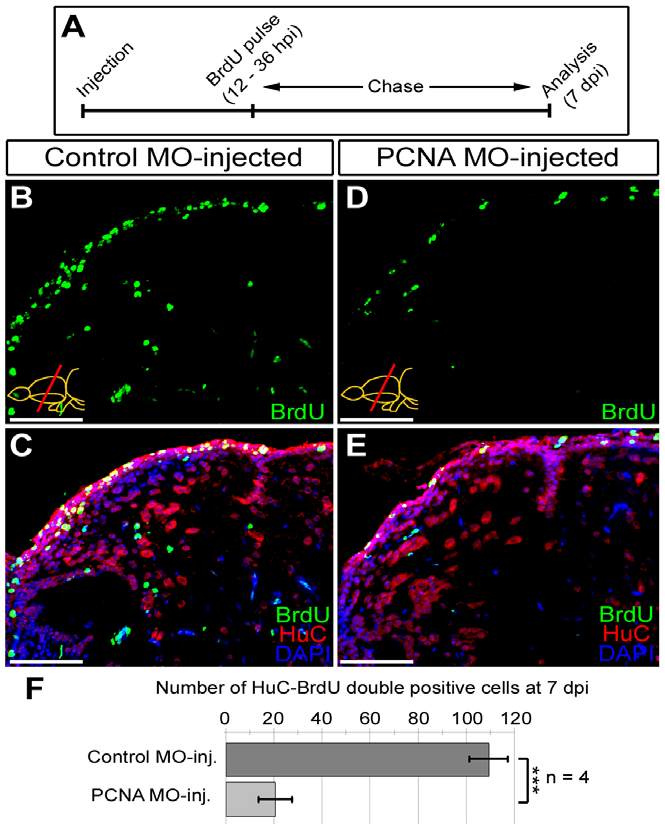
Knocking-down PCNA reduces neurogenesis as a functional consequence. (A) Schematic representation of the neurogenesis assay after PCNA knock-down. Following morpholino injection (control and PCNA-antisense), BrdU is given between 12 and 36 hours. Brains were analyzed at 7 days post injection (dpi) for BrdU (marker for proliferated cells), HuC (neuronal marker) and DAPI as a nuclear counterstain. (B) BrdU immunohistochemistry on the dorsal region of telencephalon from control morpholino-injected brain. (C) Co-staining for HuC and BrdU in control morpholino-injected dorsal telencephalon. (D) BrdU immunohistochemistry on the dorsal region of telencephalon from PCNA morpholino-injected brain. (E) Co-staining for HuC and BrdU in PCNA morpholino-injected dorsal telencephalon. (F) Graph depicts the average number of newborn neurons (HuC-BrdU double-positive cells) in control and PCNA morpholino-injected brains. Scale bars 50 µm. N = 4 adult fish.

In order to determine whether the knockdown with CVMI results in functional consequences, we injected PCNA morpholino to adult fish brains and labeled the cycling cells between 12 hpi and 36 hpi by BrdU administration, which is followed by a chase period until 7 dpi (Fig. 5A). After BrdU (labels proliferated cells during the administration period) and HuC (labels neurons) double immunostainings, we observed that compared to the control morpholino-injected brains (Fig. 5B, 5C), PCNA morpholino-injected brains significantly reduce the number of newborn neurons (Fig. 5D, 5E), which amounts to 81.2±9.7% (Fig. 5F). These results indicate that gene knockdown using CVMI in adult zebrafish brain is useful for functional studies to analyze the role of individual genes.

### Knockdowns with CVMI is dose-dependent

**Figure 6:**
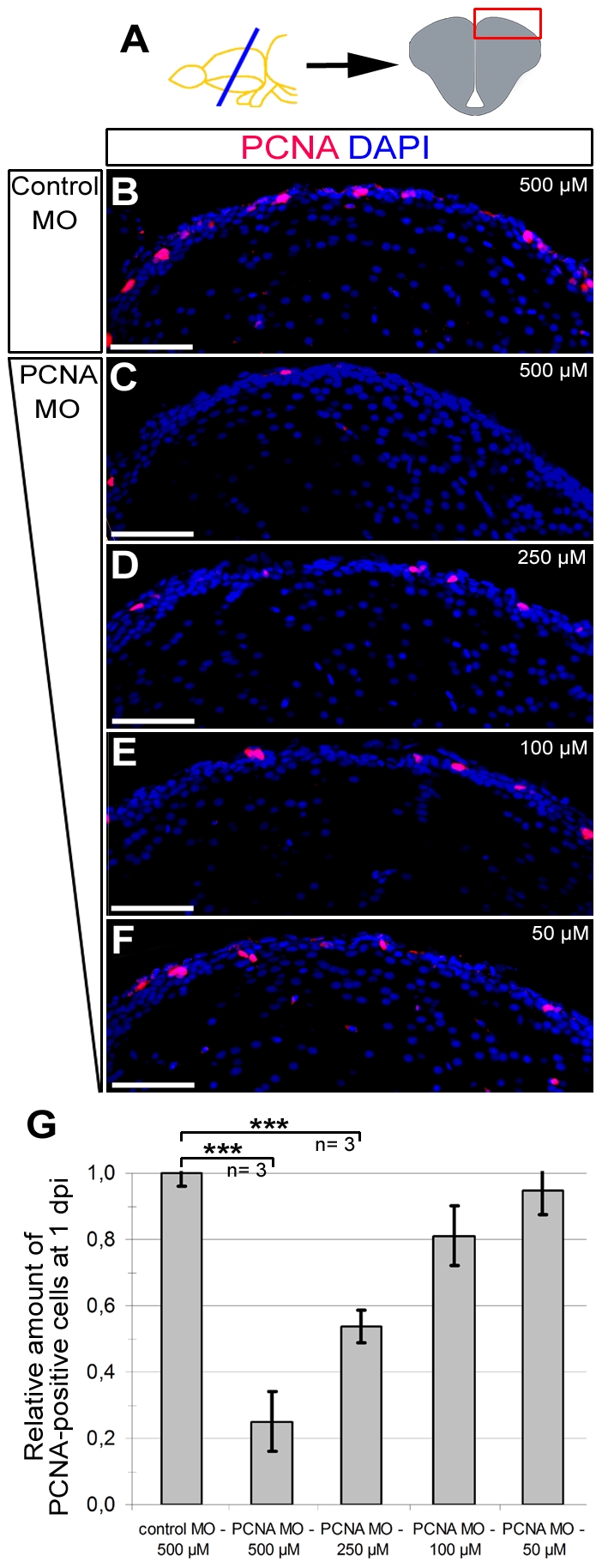
Morpholino-mediated gene knock-down using CVMI is dose-dependent. (A) The dose-response analyses were performed on dorsal regions of telencephalic sections. PCNA immunohistochemistry coupled to DAPI nuclear counterstaining on 500 µM control morpholino-injected (B), 500 µM PCNA morpholino-injected (C), 250 µM PCNA morpholino-injected (D), 100 µM PCNA morpholino-injected (E), 50 µM PCNA morpholino-injected (F) brains. (G) Graph depicts the relative amount of PCNA-positive cells in dorsal telencephalon after every dose in control and PCNA morpholino-injected brains. Scale bars 50 µm. N = 3 adult fish for every dose.

To analyze the effect of injected dose of vivo morpholinos on the extent of knockdowns, we injected different doses of PCNA morpholino to adult zebrafish brains and analyzed them at 1 dpi (Fig. 6), which is the time point when maximum efficiency is attained (Fig. 4S). We observed that compared to control morpholino injections (Fig. 6B), knockdown with PCNA antisense morpholinos leads to a dose-dependent knockdown (Fig. 6C–6F) as lesser concentrations of morpholinos results in poorer levels of knock-down (74.6±8.2% at 500 µM, 46.4±5.2% at 250 µM, 19.7±7.9% at 100 µM and 4.3±4.2% at 50 µM; Fig. 6G). These results suggest that the concentration of the morpholinos to be injected using CVMI should be optimized according to the needs. Moreover, the dose-dependency of knockdowns suggests that it is also possible to mimic hypomorphic conditions with the CVMI method.

### *Fgf8* injection

**Figure 7:**
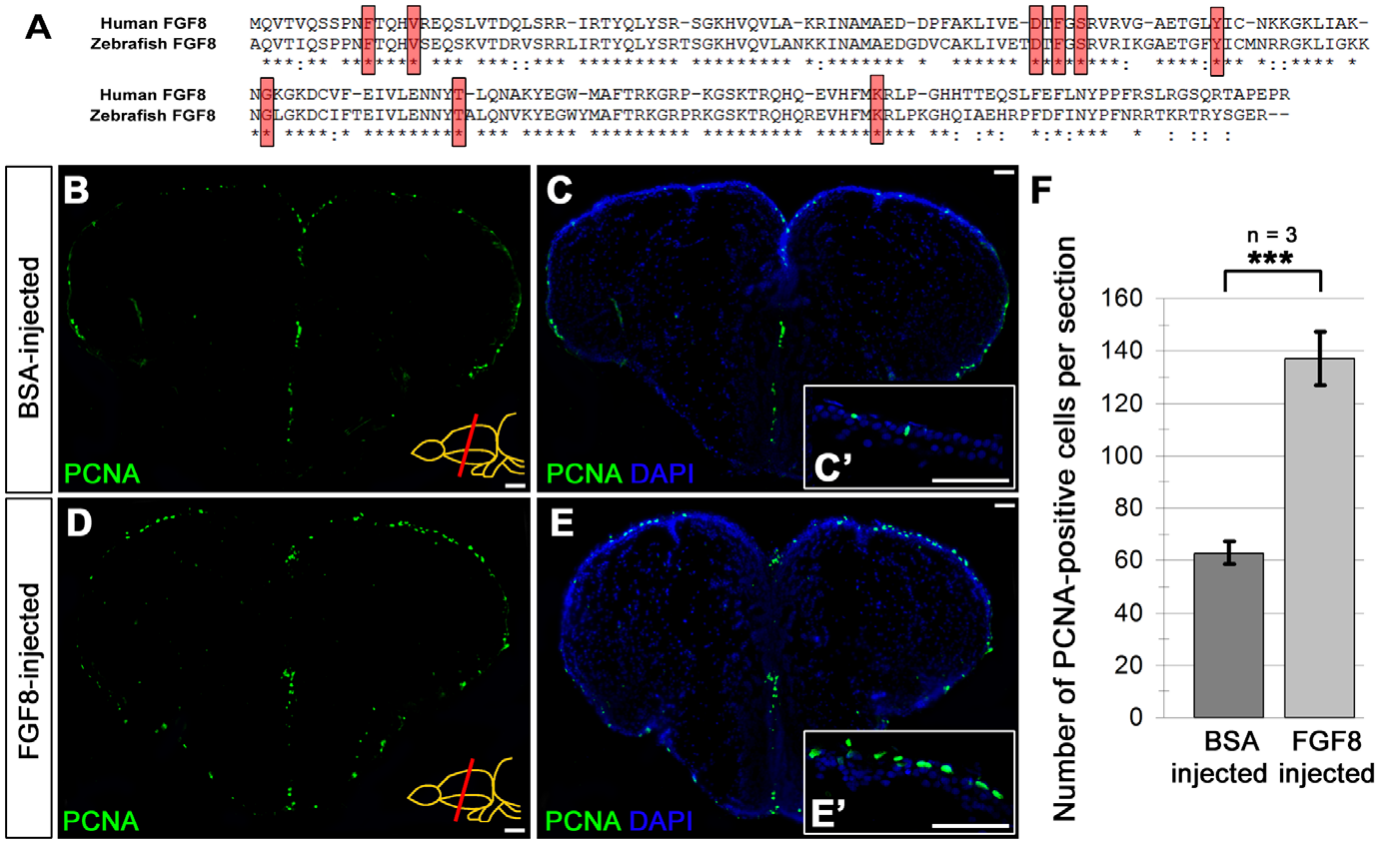
CVMI of FGF8 increases ventricular cell proliferation. (A) Pairwise sequence alignment between human FGF8 (NP_149355) and zebrafish Fgf8 (NP_571356) showing 86.4% similarity between two proteins. Red boxes indicate the residues required for ligand-receptor interaction and they are completely conserved between human and zebrafish. Asterisks indicate identical residues; semicolons indicate conservative substitutions. (B) PCNA immunohistochemistry (IHC) on the telencephalon of BSA-injected brains at 1 dpi. (C) DAPI counterstaining on A. (C') High magnification of dorsal telencephalon. (D) PCNA immunohistochemistry (IHC) on the telencephalon of FGF8-injected brains at 1 dpi. (E) DAPI counterstaining on C. (E') High magnification of dorsal telencephalon. (F) Graph depicts the average number of PCNA-positive cells per section. Scale bars 50 µm. N = 3 adult fish for each injection.

Since our data showed that CVMI is an efficient way of delivering compounds into the cerebroventricular fluid of the adult zebrafish brain, we tested whether CVMI could also be used for agonist-based modulation of signal transduction. Previously, overexpression of *fgf8* using a heat-inducible transgenic line was shown to increase cell proliferation in the ventricular region of the adult zebrafish telencephalon [5]. Therefore, we injected recombinant human FGF8 protein to adult fish brain in order to see whether administration of FGF8 protein would modulate the cell proliferation levels (Fig. 7). Human FGF8 (GeneBank Accession ID: NP_149355) is 86.4% similar, and 76.3% identical to zebrafish Fgf8 (GeneBank Accession ID: NP_571356) in its core sequence (Fig. 7A). Additionally, the nine residues important for human FGF8 ligand-receptor binding [41] are all conserved in zebrafish (Fig. 7A, red boxes), suggesting that human FGF8 is likely to be functional also in zebrafish. After injections, we observed that compared to control injections (Fig. 7B–7C'), FGF8 increases the PCNA positive cells at the ventricular region of the telencephalon by 226.2±29.6% (Fig. 7D–7F). These results are similar to the transgenic overexpression of *fgf8* [5] and indicate that in addition to morpholinos, ligands can also be injected using CVMI to alter the signal transduction pathways in the ventricular cells of the zebrafish brain.

### Conclusion

We have established a cerebroventricular microinjection (CVMI) paradigm for easy and rapid introduction of morpholinos and proteins into the cerebroventricular fluid of the adult zebrafish brain. We showed that these injections efficiently knockdown gene expression or alter signaling pathways with overt functional consequences – in our case alteration of ventricular cell proliferation and subsequent neurogenesis.

CVMI is not time consuming when compared to conventional transgenic approaches, which require considerable time to generate the desired transgenic insertions driving satisfactory levels of expression in the first place. CVMI of morpholinos is preferable as it leads to a uniform knockdown while focal injections or electroporation result in mosaic targeting of cells [2]. In this study, we used morpholinos and proteins, yet CVMI may also allow administration of different drugs or other molecules that can exert a modulatory effect on cell physiology. Assaying dose responses of a single chemical or combination of chemicals are possible with CVMI, as we have shown for PCNA knockdown in this paper. By simply mixing the morpholino solutions, multiple genes may be knocked down at the same time with CVMI. Finally, this method does not compromise the survival of the animals and does not exert toxic effects on the brain. With these features, CVMI of morpholinos and proteins into adult zebrafish brain is a novel, quick and efficient way for functional studies in the ventricular cells of the adult zebrafish brain. We believe this method is useful for functional analyses of progenitor cell proliferation, molecular regulation of stem cell maintenance, neurogenesis, and regeneration response of the adult zebrafish brain.

## Materials and Methods

### Ethics statement

All animal experiments were carried out in accordance with the recommendations and permits of the Landesdirektion Dresden (Permit numbers: AZ 24D-9168.11-1/2008-2, 4 and 14). All surgery was performed under anaesthesia, and all efforts were made to minimize suffering. Fish were raised and kept at 28°C under a 14 hour light, 10 hours dark cycle, and fed with brine shrimp artemia daily as described [42].

### Cerebroventricular microinjections and knockdowns

Cerebroventricular microinjection (CVMI) consists of generating an opening through the skull to the cerebroventricular fluid (CVF) of the adult zebrafish, e.g. at the level of optic tectum and injection into the CVF (Fig. 1A). For this purpose, the fish is anaesthetized in 0.1% MESAB in a petri dish and using the sharp edge of a fine needle with a short bevel (Cat Nr: 305178, BD Biosciences) (Fig. 1B), the cranial bone close to the midline above the optic tectum is incised (Fig. 1C). This incision generates a slit through the skull with a diameter of approximately 200 µm without damaging the optic tectum underneath (Fig. 1D). A maximum of 500 nl of solution is injected using thin glass capillaries (TWF10, World Precision Instruments) (Fig. 1E). We used the following parameters for the injection: hold pressure 20 psi, eject pressure 10 psi, period value of 2.5 and 100 ms range of gating. Injected liquid disperses through the CVF rostrally to the forebrain as determined by injection of a cell tracker dye (50 fmol, CMTPX-Red, Molecular Probes) (Fig. 1F, 1G). Such an injection can target the ventricular and periventricular cells (5–6 cell diameters-wide region of the ventricle (Fig. 1H-1I', Fig. 2). The injection apparatus (Fig. 1J) contains a halogen light source with a ring illuminator for inverse illumination (Olympus KL1500 LCD, Fig. 1J1), a vacuum pump injector (PV820 Pneumatic Picopump, World Precision Instruments, Fig. 1J2), pressurized air source (Fig. 1J3), a dissecting microscope (Olympus SZX10, Fig. 1J4), a microinjection holder and needles (World Precision Instruments, Fig. 1J5). For this study, a live cell tracker dye (CMTPX-Red, Molecular Probes), control or antisense morpholinos (500 pmol), human recombinant FGF8 protein (Abcam, 500 pmol) and as a control BSA (Sigma, 500 pmol) were injected.

In order to manipulate gene function in the adult forebrain, we injected vivo morpholino oligonucleotides (Gene Tools, OR, USA). These morpholino compounds are tagged with a lipophilic moiety that leads to an efficient uptake of the oligonucleotides by the cells via endocytosis, and thus obviates the need for electroporation [26], [34]. To check the efficiency of vivo morpholinos in reducing protein production, we used two transgenic zebrafish lines: *Tg(H2A:GFP)* [43] (ubiquitously labels all the cells, Fig. 2) and *Tg(her4.1:mCherry)* [40] (labels the ventricular radial glial cells with mCherry reporter, Fig. 3); as well as wild type zebrafish for endogenous PCNA. We used standard control and translation-blocking vivo morpholinos targeted to the mCherry, GFP and PCNA translation start sites. Morpholino sequences are as follows: *mCherry* ATG-morpholino: 5′-*TCCTCCTCGCCCTTGCTCACCATGG*-3′; *gfp* ATG-morpholino: 5′-*CAGCTCCTCGCCCTTGCTCACCATG*-3′ [44]; *pcna* ATG-morpholino: 5′-*TGAACCAGACGTGCCTCAAACATTG*-3′ [45]; standard control vivo oligo: 5′-*CCTCTTACCTCAGTTACAATTTATA*-3′.

### BrdU administration, sample preparation and immunohistochemistry

Brains were fixed in 2% PFA in PBS overnight at 4°C, incubated in Sucrose (20% w/v, Merck) – EDTA (Merck w/v, 20%) for 18 hours at 4°C, and embedded in Sucrose (20% w/v) – Gelatin (7.5% w/v, Sigma) on dry ice. Brains were sliced at 14-µm thickness using a cryostat microtome. Sections were stored at −20°C for up to six months. All immunohistochemical stainings and antigen retrievals were performed as described [1], [4], [5], [30], [40]. Primary antibodies: chicken anti-GFP IgG (Abcam, 1∶750), mouse anti-PCNA (PC10) IgG_2a_ (Dako Cyto, 1∶500), rat anti-BrdU IgG_2a_ (Serotec, 1∶500), mouse anti-HuC/D IgG_2b_ (Molecular Probes, 1∶150). Secondary antibodies: goat anti chicken Alexa-488 (Molecular Probes, 1∶500), goat anti mouse IgG_2a_ Alexa-488 or Alexa-555 (Molecular Probes, 1∶500), goat anti rat IgG_2a_ Alexa-488 (Molecular Probes, 1∶500), goat anti mouse IgG_2b_ Alexa-555 (Molecular Probes, 1∶500). DAPI was used for nuclear counterstaining.

### Penetration and knockdown efficiency assays for vivo morpholinos

We determined the penetration and knockdown capacity of vivo morpholinos by GFP immunohistochemistry on control and antisense morpholino-injected brain cryosections after knocking down GFP expression in *Tg(H2A:GFP)* transgenic line (Fig. 2). We determined the depth and efficient range of knockdown relative to the ventricle by 3D surface analysis, distribution of the average line intensity, and fluorescence intensity per cell diameter using Fiji software (http://pacific.mpi-cbg.de/wiki/index.php/Downloads; Plot Profile, 3D Surface Plot and Histogram functions). Surface plot is shown as relative fluorescence intensity per cell in 3D coordinates (Fig. 2E, 2F). Efficient knockdown range is determined by overt deviation of fluorescence intensity values of GFP morpholino-injected brains compared to control morpholino-injected brains on intensity histograms (Fig. 2G). Penetration efficiency in terms of cell diameters is calculated by measuring the ratio of GFP morpholino-injected brain GFP fluorescence intensity values (FI_GFP-MO_) to control morpholino-injected brain GFP fluorescence intensity values (FI_Ctrl-MO_) (Fig. 2H). Three fish were used for analyses. We quantified the effects of vivo morpholino injection on mCherry as a reporter transgene (*Tg(her4.1:mCherry)*) and PCNA as an endogenous gene by measuring the mCherry fluorescence intensity of the radial glial cells (Fig. 3) and PCNA immunostaining on wt zebrafish brains at 0, 6, 12 hpi; 1, 3, 5, and 7 dpi (Fig. 4). We used the histogram function of the Fiji software (http://pacific.mpi-cbg.de/wiki/index.php/Downloads) for intensity measurements. Intensity values are depicted as relative values to the control morpholino-injected fish. For PCNA, we counted the PCNA-positive cells on sections and depicted the graphs as average number of PCNA-cells per section.

### Functional neurogenesis assay

In order to analyze the consequence of PCNA knockdowns, we soaked the control and PCNA antisense morpholino-injected fish at 1-day post injection (dpi) in Tübingen E3 medium containing BrdU (10 mM, Sigma) for 24 hours. We chased for BrdU incorporation until 7 dpi (Fig. 5A). We performed BrdU and HuC double immunohistochemistry and counted HuC-BrdU double positive cells on sections (Fig. 5). We depicted the graphs as average number of HuC/BrdU double-positive cells per section.

### Dose Response Assay

For dose response experiments, we injected 500, 250, 100 and 50 pmol of PCNA morpholinos (Fig. 6) and brains were fixed at 1 dpi. PCNA immunostaining was performed and the number of PCNA-positive cells were counted on the dorsal telencephalon. Graph is depicted as the relative amount of PCNA-positive cells.

### CVMI of FGF8

Human recombinant FGF8 protein was reconstituted in PBS/0.1% BSA to a final concentration of 0.2 mg/ml. In total 500 pmol FGF8 was injected. Equimolar BSA was used as control injections. Brains were fixed at 1 dpi and PCNA immunostaining was performed (Fig. 7). Graph is depicted as average number of PCNA-positive cells per section.

### Imaging and statistical analysis

Fluorescence images were taken using structured illumination microscope (Zeiss Apotome AxioImager.Z1) or laser scanning confocal microscope (Zeiss 510 META). For statistical analysis, two-way ANOVA and Tukey's post-test were used. The following star scheme is used: *: p≤0.05, **: p≤0.005, ***: p≤0.001. The number of fish used for every experiment is indicated in the corresponding figure legends.
